# The metabolic characteristics and changes of chondrocytes *in vivo* and *in vitro* in osteoarthritis

**DOI:** 10.3389/fendo.2024.1393550

**Published:** 2024-05-24

**Authors:** Miradj Siddick Adam, Huangming Zhuang, Xunshan Ren, Yuelong Zhang, Panghu Zhou

**Affiliations:** Department of Orthopedics, Renmin Hospital of Wuhan University, Wuhan, China

**Keywords:** osteoarthritis, metabolism, chondrocyte, glycolysis, oxidative phosphorylation, lipid metabolism, mitochondrial dysfunction

## Abstract

Osteoarthritis (OA) is an intricate pathological condition that primarily affects the entire synovial joint, especially the hip, hand, and knee joints. This results in inflammation in the synovium and osteochondral injuries, ultimately causing functional limitations and joint dysfunction. The key mechanism responsible for maintaining articular cartilage function is chondrocyte metabolism, which involves energy generation through glycolysis, oxidative phosphorylation, and other metabolic pathways. Some studies have shown that chondrocytes in OA exhibit increased glycolytic activity, leading to elevated lactate production and decreased cartilage matrix synthesis. In OA cartilage, chondrocytes display alterations in mitochondrial activity, such as decreased ATP generation and increased oxidative stress, which can contribute to cartilage deterioration. Chondrocyte metabolism also involves anabolic processes for extracellular matrix substrate production and energy generation. During OA, chondrocytes undergo considerable metabolic changes in different aspects, leading to articular cartilage homeostasis deterioration. Numerous studies have been carried out to provide tangible therapies for OA by using various models *in vivo* and *in vitro* targeting chondrocyte metabolism, although there are still certain limitations. With growing evidence indicating the essential role of chondrocyte metabolism in disease etiology, this literature review explores the metabolic characteristics and changes of chondrocytes in the presence of OA, both *in vivo* and *in vitro*. To provide insight into the complex metabolic reprogramming crucial in chondrocytes during OA progression, we investigate the dynamic interaction between metabolic pathways, such as glycolysis, lipid metabolism, and mitochondrial function. In addition, this review highlights prospective future research directions for novel approaches to diagnosis and treatment. Adopting a multifaceted strategy, our review aims to offer a comprehensive understanding of the metabolic intricacies within chondrocytes in OA, with the ultimate goal of identifying therapeutic targets capable of modulating chondrocyte metabolism for the treatment of OA.

## 1 Introduction

Osteoarthritis (OA) is a complex medical condition that impacts the entire synovial joint system, particularly the hip, hand, and knee joints (1, 2). It is characterized by cartilage and meniscus degradation, inflammation and fibrosis of the infrapatellar fat pat and synovial membrane, and subchondral bone remodeling (3–5). OA is the primary origin of pain-related impairment and has the highest prevalence of all types of arthritis worldwide (6). Several risk factors have been associated with the evolution of OA. These risk factors can be categorized into individual-level factors, such as age, gender, obesity, genetics, and diet, as well as joint-level factors, like injuries and abnormal joint loading (7). Age is considered the most important factor in OA (8). Although the exact mechanism causing joint damage is not well understood, it is likely due to multiple factors such as cartilage thinning, oxidative damage, muscle weakness, and decreased proprioception (9). Currently, most treatment methods used today are only successful in relieving pain instead of healing the cartilage damage (1, 10). Physical modalities, pharmacologic treatments, and surgical treatments are the main types of OA clinical therapies. Presently, non-steroidal anti-inflammatory drugs (NSAIDs), opioids, glucocorticoids, chondroprotective substances, and symptomatic and anti-cytokines are the medication classes utilized to treat OA (11). Surgical treatment is usually chosen to treat the advanced stages of OA (12).

Despite the limited therapeutic options available, OA remains a challenging disease due to the incomplete understanding of the molecular processes and pathways involved in the disease, as well as the inherent regenerative limitations of cartilage (13). Articular cartilage comprises the cartilage matrix and chondrocytes, and its function is principally maintained by normal chondrocyte metabolism (14). Chondrocyte metabolism generates energy through glycolysis, oxidative phosphorylation, and other metabolic pathways. Chondrocyte metabolism can utilize various substrates in the joint synovial fluid, ranging from simple sugars to amino acids and fatty acids (15).

In OA, biomechanical changes occur within the cartilage and chondrocytes, including cartilage degeneration, mechanical loading, and alteration in the cartilage matrix (16). This leads chondrocytes to respond with an increased generation of matrix-degrading enzymes and inflammatory mediators, contributing to the progressive deterioration of OA cartilage and joint function (16).

OA is characterized by major metabolic modifications in chondrocytes, including substantial increases in their anabolic activity (17), leading to the generation of type I and III collagens instead of the usual type II collagen (18). Furthermore, OA-affected chondrocytes may exhibit altered phenotypes due to the abnormal expression of types I and III collagen, which are typically absent in normal cartilage. This shift in collagen production away from the norm is a hallmark of OA (18, 19).

Metabolic syndromes, including obesity, impact the cellular metabolism of joint tissue cells, particularly chondrocytes (20). In OA chondrocytes, the glycolytic process undergoes a shift, with *glucose transports (GLUTs)* and multiple enzymes playing a role in the disease’s development (21, 22). In individuals with type 2 diabetes, chondrocytes express various isoforms of the *GLUT/SLC2A* glucose transporters during glycolysis (23). *GLUT1* is considered insulin-insensitive and responsible for basal glucose uptake; while *GLUT4* has a high affinity for glucose, it is responsive to insulin (24). According to previous studies, healthy human chondrocytes limit glucose uptake by destroying *GLUT1* in high glucose culture conditions, whereas chondrocytes affected by OA do not suppress *GLUT1* (25). This suggests that OA alters glucose regulation. Additionally, obesity has been shown to increase the production of oxidative stress mediators and pro-inflammatory cytokines like *IL-1β*, which can affect mitochondrial activity and glucose uptake (26). Additionally, inflammatory mediators such as *IL-1β* and *TNF-α*, along with the progression of OA disease, enhance cholesterol uptake by chondrocytes and facilitate the production of oxysterol metabolites. These metabolites trigger the expression of pro-catabolic matrix-degrading enzymes, including matrix metalloproteinases (*MMPs*) and aggrecanases (27). The metabolic syndrome can directly influence OA development by promoting the generation of pro-inflammatory and catabolic factors, as well as indirectly by interfering with autophagy and senescence (28). Therefore, the metabolism of chondrocytes is intricately linked to the treatment of OA.

This literature review’s main purpose is to summarize the metabolic characteristics and changes of chondrocytes *in vivo* and *in vitro* in OA. The review begins with a basic overview of chondrocyte metabolism in normal cartilage and describes the role of various metabolic pathways within OA chondrocytes. In addition, this review also investigates potential therapeutic targets that can modulate chondrocyte metabolism. This literature review utilizes a comprehensive search strategy that includes databases such as Pubmed, ScienceDirect, and Google Scholar.

## 2 Chondrocyte metabolism and changes during OA

In normal conditions, the chondrocytes in the articular cartilage are quiescent, showing minimal metabolic activity and matrix component turnover (29). When articular cartilage is moderately damaged, it usually involves an injury that allows a temporary aggregation of ECM molecules and chondrocytes, facilitating a brief period of cell growth and ECM formation. Chondrocytes respond to this damage by temporarily increasing their activity to repair and restore the ECM (30). Chondrocytes respond to this damage by temporarily increasing their activity to repair and restore the ECM. Following the deterioration of the articular cartilage, the capacity of chondrocytes to regenerate in a pathological state rapidly declines. Additionally, the limited blood supply to the cartilage matrix hinders its ability to recover and regain integrity (31). This process leads to poor chondrocyte vitality, exceptionally high levels of apoptosis, and ultimately an imbalance of chondrocyte metabolism, which in turn causes articular cartilage degradation and synthetic remodeling of the ECM (32).

In healthy circumstances, chondrocyte anabolism and catabolism are balanced to preserve the structure of articular cartilage. Matrix resorption is accelerated during the degenerative process, causing degradation to occur more quickly than the chondrocytes’ anabolic attempt to create a new matrix (33).

Various metabolic processes are involved in chondrocytes, such as glycolysis, oxidative phosphorylation, and lipid metabolism. These metabolic pathways maintain chondrocyte homeostasis and cartilage integrity, and their alteration contributes to cartilage degradation, inflammation, and apoptosis.

### 2.1 Glycolysis

In chondrocytes, the hypoxic environment of cartilage leads to the generation of over 75% of total cellular ATP through glycolysis and the remaining energy through oxidative phosphorylation (OXPHOS). Glucose is considered the main metabolic fuel and structural precursor in this process (2). During glycolysis, a single molecule of glucose can generate two molecules of pyruvate while producing two molecules of ATP (34). Pyruvate can then reach the mitochondria in aerobic circumstances, where pyruvate dehydrogenase complexes convert it into acetyl-CoA, thus integrating it into the tricarboxylic acid cycle (TCA). This cycle produces GTP, which is the energy equivalent of ATP, as well as NADH and FADH_2_, crucial electron carriers in the electron transport chain for oxidative phosphorylation. This process ultimately leads to the generation of ATP (35). In the cytosol, pyruvate is converted to lactate to restore the NAD^+^ levels essential for the continuous synthesis of ATP by phosphorylation of the substrate via anaerobic glycolysis (31). OA chondrocytes exhibit an elevated anaerobic glycolysis rate (36). In **Table 1**, the key targets in the pathophysiology of glycolysis-related OA and their subtypes are summarized. The metabolic function of each process and its role in OA pathogenesis are outlined.

**Table 1:** The key targets in the pathogenesis of glycolysis-related OA.

Targets	Subtypes	Metabolic role	Role in OA pathophysiology
Glucose transporters	GLUT-1	Responsible for glucose transport to the cell	Transport glucose in chondrocytes, when *GLUT-1* is upregulated induces cartilage damage by enhancing glucose absorption and creating excessive AGEs (37–39)
Pyruvate kinase	PKM2	Generates ATP by converting phosphoenolpyruvate to pyruvate	Inhibiting *PKM2* can limit OA chondrocyte growth, induce cell apoptosis, and diminish *COL21* and *SOX9* expression levels (2)
Phosphofructokinase	PFKFB3	Essential for glycolysis stimulation	Increases chondrocyte cell vitality, inhibits caspase-3 activation and stimulates the production of aggrecan and type II collagen (40)
Lactate dehydrogenase	LDHA	Yields lactate from pyruvate	Enhances ROS production in chondrocytes in inflammatory condition (40)

In OA chondrocytes, the glycolytic process undergoes a shift, and the *GLUTs* and multiple enzymes are considered to be involved in the pathogenesis. Various types of the *GLUTs* family, including *GLUTs*-1, *-3*, *-6*, *-8*, *-9*, and *-10*, have been identified in human chondrocytes through protein analysis (21, 22). *GLUT-1* is essentially responsible for basal glucose transfer in chondrocytes, glycolysis’s first rate-limiting phase (41), which is indispensable for chondrogenesis, embryonic development, and skeletal system development (42). However, the expression of *GLUT-1* is increased during hypoxia and glucose deficiency but downregulated in high-glucose circumstances (40). When glucose levels increase, chondrocytes that cannot adapt may exhibit heightened glucose absorption and subsequently generate elevated levels of reactive oxygen species (40). The disruption of cellular growth and matrix synthesis in the growth plate and articular cartilage occurs due to *GLUT1* gene deletion, leading to long-term bone dysplasia and cartilage fibrosis (43, 44). Conversely, prolonged elevation of *GLUT1* expression can detrimentally impact cartilage by promoting excessive glucose absorption and the accumulation of advanced glycation end-products (37–39). Thus, in the presence of pro-inflammatory cytokines, chondrocyte *GLUT1* expression is remarkably elevated (41), suggesting that *GLUT-1* may serve as a potential therapeutic target to treat OA.

Glycolysis is a highly regulated process wherein a multitude of enzymes play relevant roles (45). These enzymes include *hexokinase (HK), pyruvate kinase (PK), phosphofructokinase (PFK)*, and *lactate dehydrogenase A (LDHA)*. The subtype *HK2* is an important regulator that facilitates the passage of glucose metabolism from oxidative phosphorylation to aerobic glycolysis (46). The glycolysis initial rate-limiting enzyme, *HK2*, is capable of catalyzing the conversion of glucose to glucose-6-phosphate (G-6-P) and is implicated in the primary glycolysis pathways (47). In OA chondrocytes, *transforming growth factor beta 1 (TGF-β1)* promotes *HK2* expression (48). Compared to the group with healthy synovial tissue, OA synovial tissue (FLS) had a greater *HK2* level of expression. In OA FLS, overexpression of *HK2* increases RNA expression levels of pro-inflammatory cytokines such as *IL-6*, *IL-8*, and *MMPs* (49). *PK* catalyzes the final and rate-limiting glycolytic process to transform phosphoenolpyruvate to pyruvate and produce ATP. The isoenzyme *PKM2* is increased while ATP generation is reduced in human OA chondrocytes. *PKM2* inhibition can limit OA chondrocyte growth, induce cell apoptosis, and diminish *COL21* and *SOX9* expression levels. *PKM2* overexpression causes lactate accumulation and creates an acidic microenvironment in OA chondrocytes. The acidic microenvironment impairs the production of chondrocyte matrix and may increase cartilage deterioration in OA (50), indicating that *PK* might play a key function in OA progression and could be an essential target to reverse OA pathogenesis.

The enzyme *PFK*, notably *Phosphofructokinase-2/Fructose-2, 6-Bisphosphatase 3 (PFKFB3)*, is important for glycolysis stimulation. When chondrocytes are activated with tumor necrosis factor (*TNF*) or *IL-1*, *PFKFB3* is decreased. In addition, *PFKFB3* can increase chondrocyte vitality, inhibit caspase-3 activation, and stimulate the expression of aggrecan and type II collagen, which could serve as a target for treating and preventing OA (51).

*LDHA* is required for lactate synthesis (52, 53). Some synovial fluid studies revealed that OA patients have higher levels of lactic acid in the absence of sepsis, which suggests that *LDHA* is likely to play a pathogenic role in human OA (54). *LDH* activity and expression are greatly increased in IL-1-treated primary chondrocytes. In an inflammatory condition, *LDHA* can enhance the production of ROS in chondrocytes (40). Therefore, *LDHA* could be another therapeutic vision for OA treatment.

Additionally, *glyceraldehyde 3-phosphate dehydrogenase (GAPDH)*, which is primarily responsible for glucose breakdown in glycolysis, participates in a variety of cell functions, such as phosphotransferase activity, RNA export, DNA replication, gene transcription activation, and gene translocation regulation. The study revealed that the expression of *GAPDH* is influenced by hypoxic conditions in 3-dimensional (3D) culture (55). This association observed in 3D culture could be related to hypoxia-induced extracellular matrix formation (55). This indicates that hypoxia might contribute to the development of OA.

Chondrocyte *GLUTs* exhibit sensitivity to mechanical pressure or loading. Pressure loading reduces glucose transport via *GLUTs*. Glucose transport in chondrocytes may also be influenced by several growth factors and cytokines. Interleukin-1, *transforming growth factor-1 (TGF-1)*, insulin-like growth factor-1, *TNF*, and others can enhance glucose absorption by chondrocytes via several pathways (56).

### 2.2 Lipid metabolism

Lipids, characterized by their intricate structures such as fatty acids, glycerol, and numerous functional groups, are among the most important molecules in biology. They are essential for maintaining biological activities by performing functions including energy storage, cell membrane structure, and signaling (57). Within the human body, there are four major types of lipids: cholesterol, fatty acids (FAs), triglycerides (TGs), and phospholipids. Despite constituting less than 1% of the wet weight of adult articular cartilage, lipids are present in both the chondrocytes and matrix (58, 59).

Several studies have found that dysregulated lipid metabolism contributes to the susceptibility to OA by promoting inflammation, cartilage deterioration, and imbalances in joint tissue homeostasis (60). Specifically, elevated blood cholesterol levels have been related to generalized OA, implying that cholesterol may contribute as a risk factor for OA (61). Moreover, abnormal HDL and higher levels of total cholesterol and TG in the bloodstream have been associated with the progression of bone marrow lesions (62, 63). Bone marrow lesions can cause discomfort and may accelerate cartilage loss in the knees of OA patients (64–67).

Dyslipidemia, particularly reduced HDL levels, can impair cartilage homeostasis and contribute to OA progression (63). Decreased HDL levels result in cholesterol accumulation within the cartilage tissue, compromising the regular metabolic functions of chondrocytes and the structural integrity of the extracellular matrix. This may lead to low-grade inflammation, oxidative stress, and cartilage deterioration (68, 69). Therefore, controlling dyslipidemia and maintaining normal HDL levels may prevent or reduce the progression of OA.

However, in osteoarthritic chondrocytes, important lipid deposit reserves have been noted (58). A positive correlation exists between the gravity of OA and the quantity of intracellular lipid deposit reserves (58). The downregulation of cholesterol efflux genes such as *APOA1* and *ABCA*1 in OA cartilage contributes to lipid buildup in chondrocytes. This disruption of cholesterol metabolism may disturb the normal lipid balance within the joint, resulting in increased inflammation and oxidative stress, both of which are known to contribute to the progression of OA. Thus, the impaired expression of lipid-regulating genes appears to have a significant impact on the etiology of OA by contributing to lipid buildup and its negative effects on cartilage health (70). According to the research of Lippiello (1991), the distribution profile of individual fatty acids in both healthy and osteoarthritic cartilage was kept at a specific level, with 85% of the total fatty acids being composed of palmitic, oleic, and linoleic acids. The study also revealed that there were no changes in cholesterol content. However, OA samples had significantly greater levels of total fatty acids and arachidonic acid, and these raised levels were linked to increasing histological severity (58). Baker (2012) discovered in the Multicenter OA Study that there is a positive correlation between synovitis and omega-6 PUFA, suggesting that higher omega-6 PUFA intake may worsen inflammation in OA. In contrast, they found an adverse association between total omega-3 PUFA levels and patellofemoral cartilage loss, indicating that a higher intake of omega-3 PUFA may have a preventive effect on cartilage health in OA (71). These findings suggest that maintaining a balance of omega-3 and omega-6 PUFA in the diet may influence the progression of OA.

**Figure 1:**
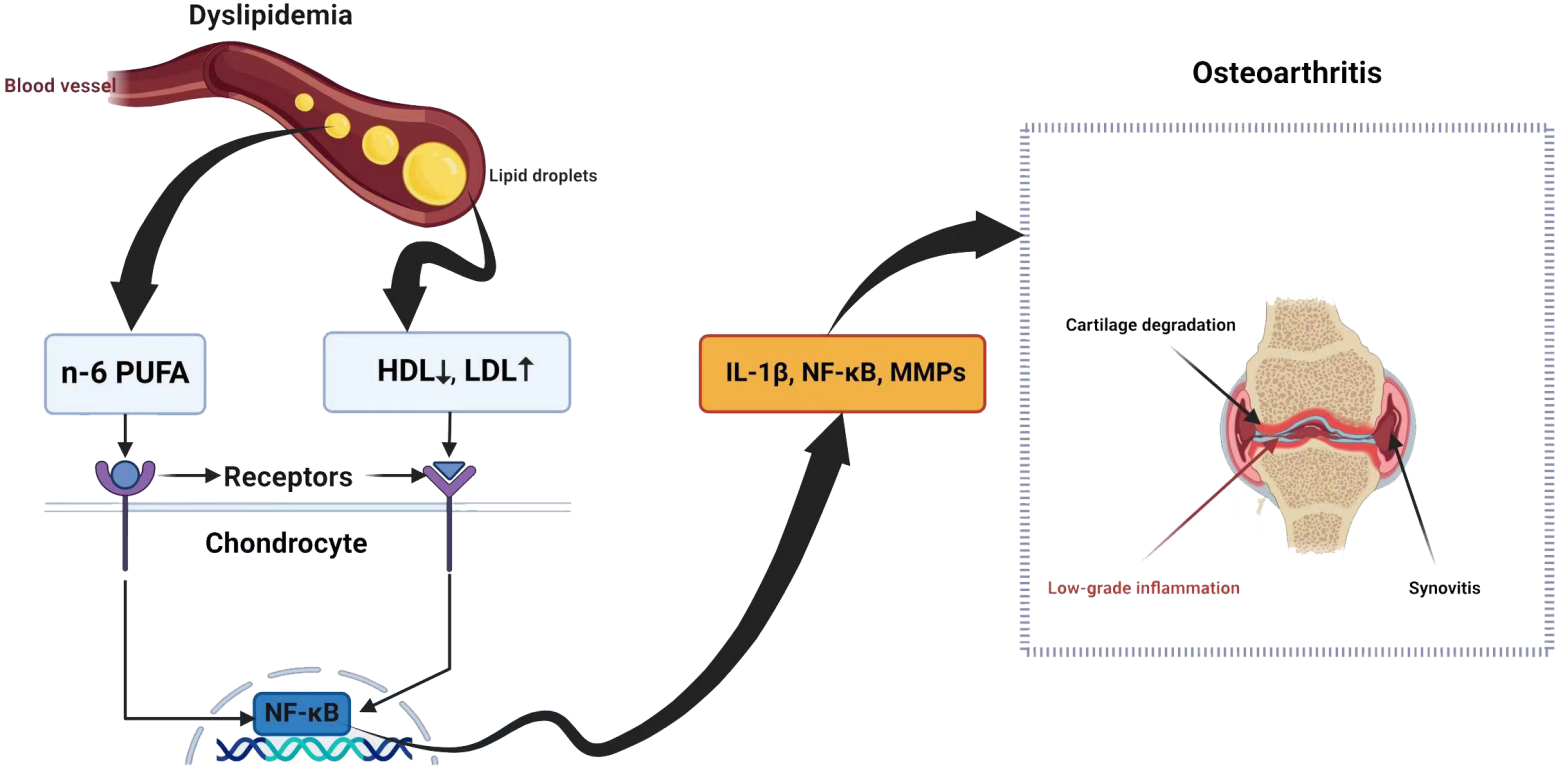
Dyslipidemia and osteoarthritis share a complex relationship with potential implications for joint health.

The synovium consists of macrophages, fibroblasts, and endothelial cells (72). In OA, these cells can trigger the release of cartilage substances such as *IL-1* and *TNF-α*, which generate *MMPs* and inhibit the production of collagen and proteoglycan, leading to low-grade synovitis (73). Inflammatory conditions may result in LDL oxidation, which is subsequently absorbed by synovial cells via scavenger receptors (72). An elevated level of LDL could trigger synovial cell activation, potentially resulting in increased synovial thickness (72). Thus, lipid metabolism is related to synovitis during OA (**Figure 1**).

Also, cholesterol levels in OA chondrocytes are higher due to the increased synthesis of oxysterol metabolites and the activation of cholesterol hydroxylase (27).

#### 2.2.1 Hypercholesterolemia

Hypercholesterolemia exacerbates OA via various mechanisms. Several studies have confirmed a link between higher levels of cholesterol oxidation products (oxysterols) and mitochondrial-derived oxidative stress, which in turn generally increases mitochondrial reactive oxygen species (mtROS) (74). Therefore, higher levels of cholesterol oxidation products can disrupt mitochondrial function, leading to increased ROS production and oxidative stress, ultimately contributing to cellular dysfunction and disease development (75). In normal conditions, enzymes such as superoxide dismutases (SODs) and glutathione peroxidase (GPX) normally remove ROS (76). Human chondrocytes regularly produce GPX, cytosolic Cu/Zn, and mitochondrial SOD. The SODs convert superoxide to hydrogen peroxide, which is removed by GPX and catalases (76). Increased ROS can surpass the protective mechanisms against oxidative stress in mitochondria, causing irreversible harm to sensitive cells and permanent damage to adjacent tissues. The excessive production of ROS, especially superoxide and related radicals, has been linked with cellular failure in both human and animal studies (77). Meanwhile, ROS overproduction is associated with human cartilage dysfunction (78).

Mito Tempo, a targeted antioxidant, reduces oxidative stress in cells by scavenging mitochondrial superoxide, which helps prevent the generation of harmful ROS like cytosolic hydrogen peroxide (79). This mechanism may alleviate OA symptoms caused by hypercholesterolemia, allowing cells to eliminate excess oxidative stress (77), suggesting that mitochondrial-targeted antioxidants are a promising therapy for OA-caused hypercholesterolemia (80). Studies have demonstrated that Mito Tempo treatment can reduce cartilage degradation, inhibit inflammatory cytokines production, and improve joint function in OA animal models (29). However, further clinical trials are needed to determine the complete effectiveness of Mito Tempo in treating OA.

*ABCA1*, an ATP-binding cassette transporter, is a plasma membrane protein that removes excess free cholesterol and phospholipids from the tissues (81, 82). This process involves transporting cellular cholesterol and phospholipids to lipid-free apolipoprotein AI, leading to the generation of nascent HDL particles (81, 82). Furthermore, recent investigations highlight the significant connection between inflammation and cholesterol homeostasis in the context of OA. *ABCA1* emerges as a crucial player in these pathways, influencing OA progression through its impact on inflammation and joint tissue health (83).

Overall, hypercholesterolemia influences oxidative stress and inflammation in OA by promoting the production of ROS, increasing levels of inflammatory mediators, and disrupting lipid metabolism (84). These interconnected mechanisms contribute to the progression of OA in patients with elevated cholesterol levels. However, these mechanisms need further investigation to suggest a therapy.

#### 2.2.2 Fatty acids

The variations in fatty acids (FAs), including polyunsaturated fatty acids (PUFAs), monounsaturated fatty acids (MUFAs), and saturated fatty acids (SFAs) (85), may affect the inflammatory responses caused by FAs. These responses are critical to the progression of OA (86). In chondrocytes, *TLR-4* is the most expressed *TLR* subtype (87). FAs such as SFAs can activate *TLR-4-mediated* inflammatory responses, which, via its downstream molecule myeloid differentiation factor (MyD)88, activate the NF-kB pathway, leading to the production of inflammatory cytokines (88). Additionally, SFAs activate inflammasomes in immune cells and elevate *IL-1β* (89), ultimately contributing to OA progression (90). Targeting these pathways may provide potential therapeutic strategies for preventing and treating OA.

Substantial evidence shows that FAs are generally pro-inflammatory (91, 92). They stimulate adipose tissue macrophages to release *TNFα* and *IL-1* (93). In addition to SFAs, omega-6 polyunsaturated fatty acids (n-6 PUFAs) exhibit a pro-inflammatory influence. N-6 PUFAs not only favor ROS generation and chondrocyte death via the NADPH oxidase 4 (NOX-4) signaling pathway, but they can also be converted into bioactive substances such as pro-inflammatory prostaglandins and leukotrienes, which are essential in joint inflammation, the breakdown of cartilage matrix, and bone resorption in OA (48, 94–96). Compared to SFAs and n-6 PUFAs’ pro-inflammatory effects, n-3 PUFAs abrogate inflammation. For example, the interaction between n-3 PUFAs and G-protein coupled receptor 120 (GPR 120) results in the creation of protectins and resolvins, which mediate anti-inflammatory actions in several types of cells (97, 98).

Fatty acids contribute to promoting OA through several mechanisms, with the production of pro-inflammatory molecules being one of the most significant factors.

### 2.3 Mitochondrial dysfunction

To carry out their normal activity, chondrocytes need to be supplied with energy (99). The mitochondria produce ATP via the TCA, also known as the Krebs cycle or citric acid cycle, and OXPHOS (99). OXPHOS is essential for ATP generation in chondrocytes. The electron transport chain (ETC) establishes an imbalance of protons across the inner mitochondrial membrane (IMM) and generates mitochondrial membrane potential, which leads to complex V (also known as ATP synthase) producing ATP (100). Additionally, several protein complexes located within the inner mitochondrial membrane promote the movement of electrons and the pumping of protons along the mitochondrial respiratory chain to produce ATP (80). These complexes include NADH dehydrogenase (complex I), succinate dehydrogenase (complex II), Cyt-C reductase (complex III), and Cyt-C oxidase (complex IV). Mitochondrial dysfunction in OA can result in decreased activity of respiratory chain complexes I, II, III, and V, loss of *MMP*, and decreases in OXPHOS (101), leading to inflammation and IL-1β production (102).

#### 2.3.1 Reactive oxygen species production

**Figure 2:**
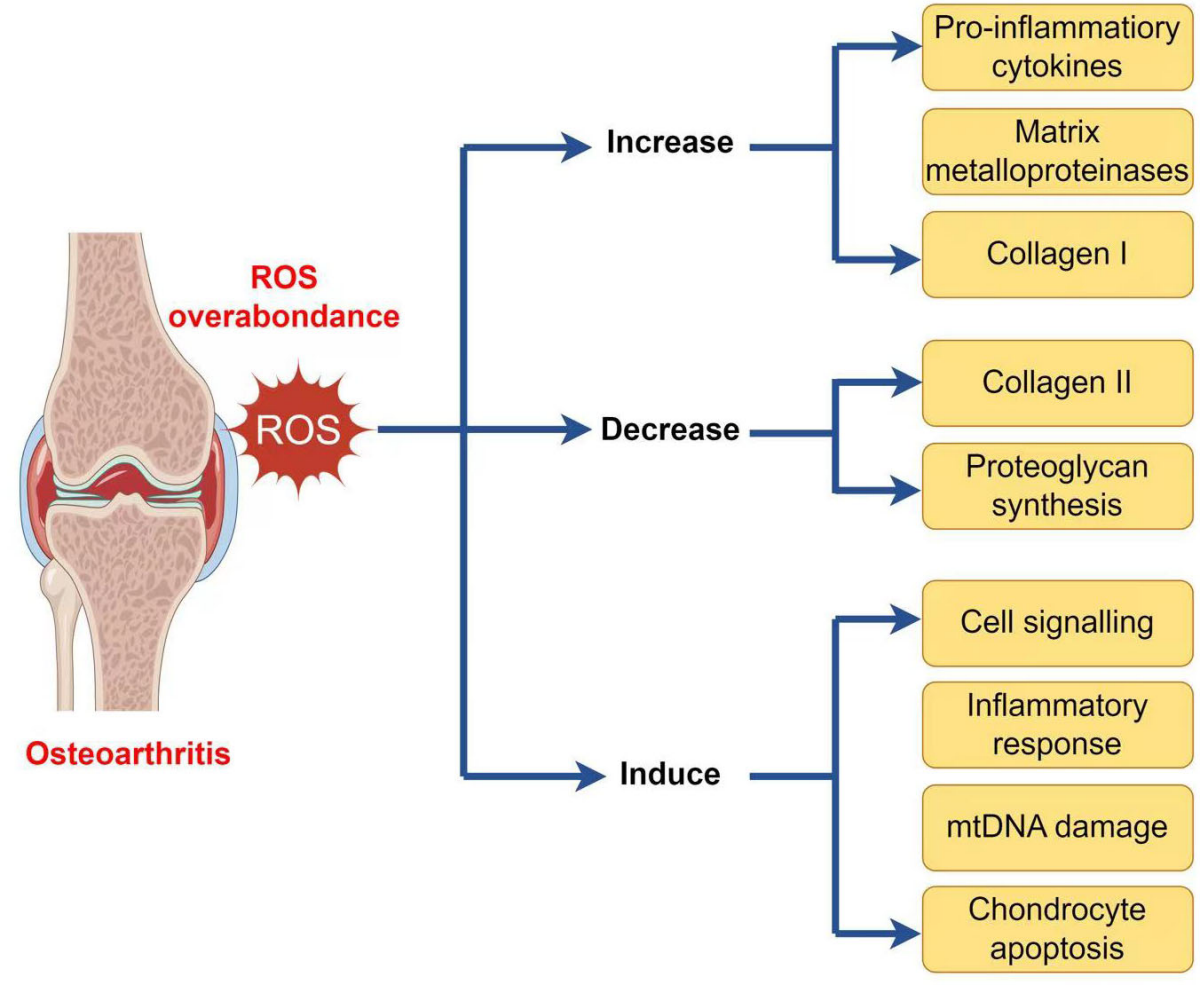
ROS implication in the pathophysiology of osteoarthritis.

As well as generating ATP, mitochondria exercise a crucial function in other physiological processes within the cells, including the generation and modulation of ROS, the detection and regulation of hypoxic conditions by *hypoxia-inducible factor-1 (HIF-1)*, mitochondria-mediated apoptosis, and the accommodation of intracellular calcium ions (103–107). Moreover, mitochondria play a crucial function in the pathophysiology and development of OA. Therefore, an overabundance of ROS can lead to both oxidative damage and involvement in redox-regulated cell signaling pathways like Akt and MAPK signaling (29). To maintain the balance of the cellular redox reaction, ROS is a sensitive signaling element of cell physiology. Signaling pathways like mitogen-activated protein kinase/extracellular signal-regulated kinase (MAPK/ERK) and insulin phosphatidylinositol-3-kinase-protein kinase B (PI3K/Akt) are triggered by excessive ROS (108). ROS may stimulate the MAPK/ERK pathway by oxidizing and activating upstream kinases, including Raf, MEK, and ERK. ROS can potentially activate the PI3K/Akt pathway by oxidizing and inhibiting phosphatases that generally inhibit Akt activation. Furthermore, ROS can activate signaling pathways through cysteine residues in key signaling molecules, causing conformational changes and activating downstream targets. Additionally, ROS can influence signaling pathways by activating transcription factors such as NF-kB and AP1, which regulate gene expression for cell survival and growth (109). ROS disrupts the production of glycosaminoglycans and type II collagen fibers while increasing the expression of collagen type I, matrix metalloproteinases, and pro-inflammatory cytokines via the MAPK and MAPK/ERK signaling pathways (110). ROS activates the PI3K/Akt and caspase pathways and can lead to chondrocyte apoptosis during the early stages of OA (111). In addition to affecting chondrocyte function, an overabundance of ROS induces *mitochondrial DNA (mtDNA)* damage and can also decrease the *mtDNA* repair capacity (**Figure 2**) (112–114).

#### 2.3.2 Mitochondria and the inflammatory response

**Figure 3:**
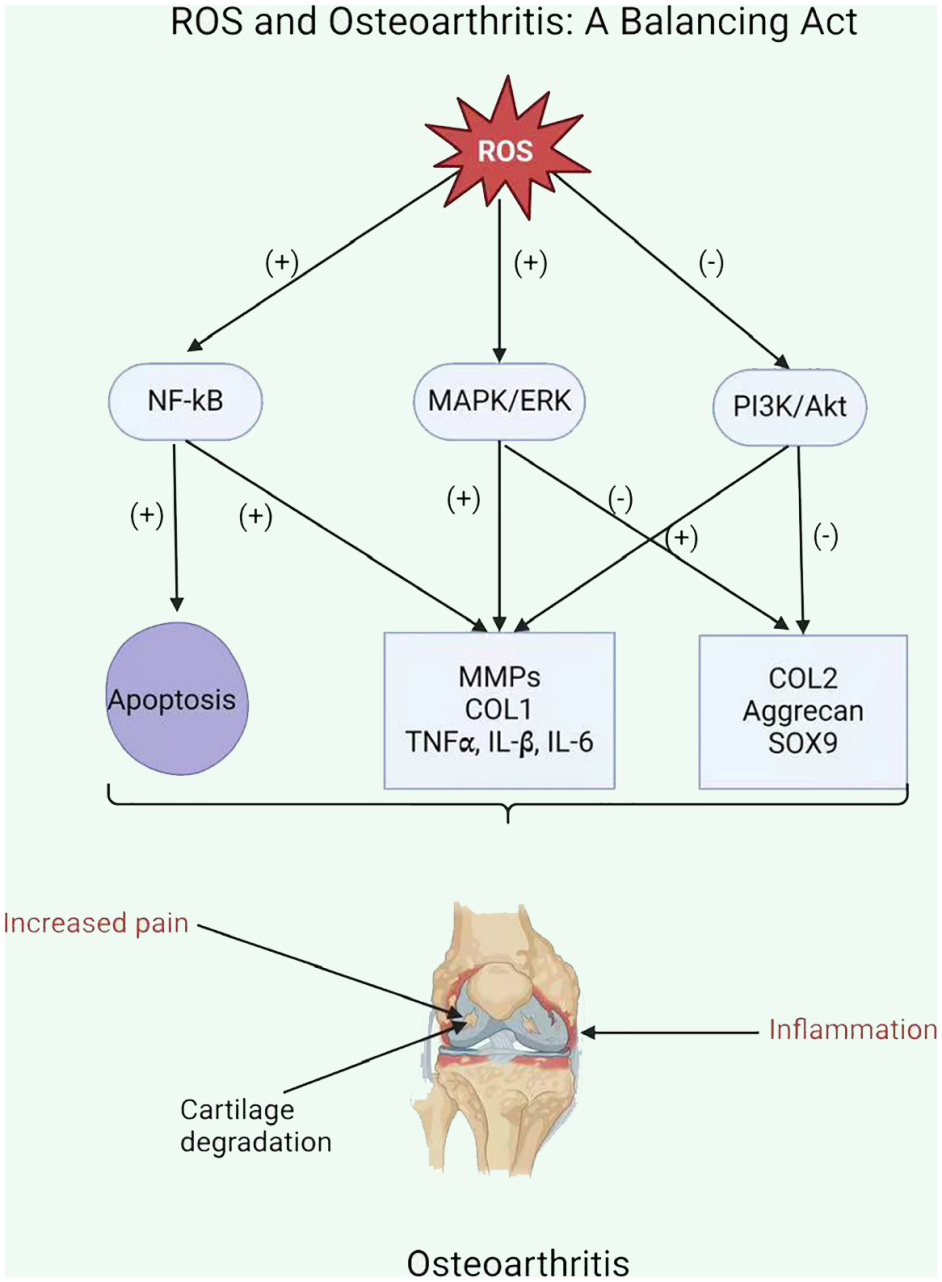
ROS and Osteoarthritis: A Balancing Act.

ROS enhances the inflammatory response in OA by stimulating signaling pathways, including NF-kB, MAPK, and PI3K/Akt (111). This stimulation upregulates pro-inflammatory cytokines (including *TNFα, IL-1β*, and *IL-6*) and *MMPs*, which damage the cartilage matrix (**Figure 3**) (115). Moreover, ROS increases the expression of tissue inhibitors of *MMPs (TIMPs)*, which function as endogenous *MMP* inhibitors (116). An imbalance among *MMPs* and *TIMPs* leads to increased extracellular matrix breakdown in OA chondrocytes. Ultimately, ROS-mediated stimulation of these pathways leads to inflammation and *MMP* overexpression in OA chondrocytes, resulting in cartilage breakdown and OA progression.

OA leads to increased ROS synthesis by chondrocytes in cartilage. The mitochondrial respiratory chain (MRC) is a significant source of ROS production (117). ROS exacerbated the inflammatory response and upregulation of matrix metalloproteinases in OA chondrocytes due to the reduced activity of mitochondrial complexes II and III (118, 119). In addition, inhibiting complexes III or IV in these cells can cause the generation of various pro-inflammatory stimuli such as cytokines IL-1, *IL-6*, and *IL-18*, prostaglandin e2 (PGe2), chemokines *IL-8* and monocyte chemotactic protein 1, and proteases *MMP1*, *MMP3*, and *MMP13* (26, 120).

#### 2.3.3 Apoptosis and cell death

Furthermore, whenever mitochondria change their morphology, structure, or function, chondrocytes manifest themselves in a pathogenic state. Increased oxidative stress, chondrocyte apoptosis, inflammation-mediated matrix breakdown, and calcification of the cartilage matrix have been observed (121, 122). The deterioration of mitochondrial activities and quality control is a major cause and characteristic of chondrocyte senescence and apoptosis, as well as the development of OA (123, 124). Oxidative stress provokes chondrocyte death via the caspase-dependent and caspase-independent mitochondrial pathways in OA (125). The mitochondria of OA chondrocytes displayed apoptosis-associated structural changes, as well as a decrease in mitochondrial membrane potential (ΔΨm) and mitochondrial respiratory chain (MRC) enzyme activity (126).

## 3 *In vivo* and *in vitro* studies of chondrocyte metabolism in OA

To shed light on *in vivo* and *in vitro* studies of glycolysis and lipid metabolism, this review is based on the cases of diabetes-related OA and obesity-related OA. Thus, this will provide us with a broad understanding of the *in vivo* and *in vitro* studies of chondrocyte glycolysis and lipid metabolism and their relationship with diabetes and obesity, given that these are major factors in the pathogenesis of OA.

### 3.1 Diabetes-related OA

Given the importance of glycolysis in chondrocyte metabolism, multiple researchers have concentrated on its exposure in high-glucose environments. Therefore, the ability of normal chondrocytes to respond to normal glucose levels is lost during OA, which is the absolute cause of excessive glucose absorption and possible glucose poisoning (25). Exposure to high glucose levels can lead to localized toxicity in joint tissue, causing an increase in oxidative stress, cytokines, proteolytic enzyme synthesis, and the accumulation of advanced glycation end products (AGEs) (127–129). Unfortunately, there is a scarcity of qualified research studies in animal models of diabetes that are related to OA. In a recent study, cartilage injury was seen after eight weeks of hyperglycemia in mice with streptozotocin (STZ)-induced type 1 diabetes, and there were higher levels of circulating AGEs (130). Pioglitazone, a diabetes medication, improved both anomalies. The authors concluded that the medication’s response indicated a downregulation in *PPARγ* expression; however, it was not so clear whether this was connected to hyperglycemia improvement or *PPARγ* suppression (131). A noteworthy investigation employed the diet-induced-obesity (DIO) model on the C57Bl/6 strain, meticulously assessing physiological parameters and histological effects related to OA (132). This study related a high-fat diet (60% calories) with the meniscal ligament damage paradigm to produce OA. The mice group that obtained both the high-fat diet and ligament injury had higher OA scores (increased joint degradation). However, because hyperglycemia was not developed until the final month of the experiment, it is uncertain whether hyperadiposity (133) or increasing hyperglycemia was the main factor behind increased OA development. This essential study describes that acceleration of joint degradation is related to metabolic abnormalities commonly observed in diabetes mellitus (DM) patients (131). Increased proteoglycan degradation has been found in the non-articular connective tissues of diabetic animals (134). The composition of cartilage’s ECM significantly affects its biomechanical qualities. Furthermore, there is some evidence that metabolic problems related to diabetes affect the cartilage ECM. Previous experiments in animal models of diabetes demonstrated decreased collagen formation (135) and enhanced proteoglycan degradation. Thus, these works show glycolytic metabolism alterations in chondrocytes in diabetes-related OA.

In an *in vitro* study, Laiguillon (2015) used diverse methods such as (14C)-2-deoxyglucose to assess glucose uptake, quantitative RT-PCR, and ELISA/EIA to observe the expression and release of pro-inflammatory mediators, and ROS and nitric oxide (NO) production were measured. Under high-glucose conditions, they showed enhanced articular chondrocyte glucose absorption, specifically in response to *IL-1β* stimulation (127). *IL-1β* has been shown to promote *GLUT-1* and *GLUT-9* production in chondrocytes (21). They obtained comparable results for *GLUT-1*. Under ordinary glucose circumstances, *IL-1β* only moderately promoted glucose absorption despite increased *GLUT-1* expression. This absorption was abundantly boosted when cells were generated under high glucose conditions (127). Also, in another study, an author observed that OA-affected chondrocytes exposed to high glucose levels were unable to downregulate *GLUT-1*, resulting in higher glucose accumulation inside the chondrocytes and the production of more ROS, which is deleterious to the ECM (136), which leads to mitochondrial dysfunction and cartilage degradation. Excessive oxidative stress may also result from the crucial cellular transfer of glucose (131). Therefore, these studies proved that chondrocytes cannot downregulate glucose concentration under high glucose conditions, but they absorb it, which may deteriorate cartilage, thus enhancing OA progression.

### 3.2 Obesity-related OA

It has been noted that OA and lipid metabolism are closely related (137, 138). Disrupted lipid metabolism is linked to obesity (139). Obesity is distinguished by high joint loading and aberrant lipid profiles, such as dyslipidemia (60). Obesity-related dyslipidemia is defined by elevated plasma levels of TGs, low levels of HDL cholesterol (HDL-c), often modestly raised levels of LDL cholesterol (LDL-c), and higher levels of FFAs (139). Previous studies have shown a potential role for HDL malfunction in the pathophysiology of OA, based on the finding that OA patients have lower serum HDL-c levels (140). To further study this, Eva Thijssen (2015) compared the development of OA in LCAT−/− and ApoA-I−/− mice and that of C57BL/6 control mice. These knockout (KO) mice had significantly lower levels of functional HDL. On the other hand, cartilage fibrillation, vertical clefts, chondrocyte clustering, and reduced levels of proteoglycan were observed in both LCAT−/− and ApoA-I−/− mice fed a Western-type diet (WTD, 42% calories from fat), while control mice did not exhibit any of these characteristics. Furthermore, KO mice on WTD showed elevated cartilage protein levels of *MMP-2*, *MMP-9*, and *MMP-13*. A decrease followed this enhanced *MMP* expression in collagen type II protein levels (60). So, all these studies support the idea that reduced HDL levels are involved in OA progression by affecting cartilage homeostasis.

Excessive levels of serum low-density lipoprotein (LDL) and oxidized LDL have been linked to pro-inflammatory effects (141). According to research by De Munter (2016), mice given a diet high in cholesterol developed synovitis, accumulated LDL in synovial cells, and produced more ectopic bone formation. They suggest that this process is caused by elevated levels of oxidized LDL activating endothelial, fibroblast, and synovial macrophages, which in turn cause ectopic bone formation, local inflammation, and cartilage loss (142). Oxidized LDL promotes OA by activating inflammatory pathways such as NF-kB and oxidative stress, leading to the generation of ROS and the expression of inflammatory mediators (*MMPs*), contributing to cartilage degradation in OA (143).

As was previously highlighted, increased systemic FFA levels are also an important factor in obesity-related dyslipidemia. FFAs can activate macrophages by engaging *Toll-like receptor 2/4 (TLR2/4)*, which results in downstream c-Jun N-terminal kinase signaling and macrophage activation (93). Consequently, pro-inflammatory mediators such as *TNF-α* can be secreted by macrophages (60). Furthermore, *TNF-α* exacerbates inflammation by stimulating the synthesis of pro-inflammatory cytokines and chemokines such as *IL-6*, *IL-8*, monocyte chemoattractant protein 1, and CC-chemokine ligand 5 (144–147). In OA, high levels of FFAs in cartilage tissues are related to significant tissue damage (58). *In vitro*, palmitate (SFA) stimulates the synthesis of pro-inflammatory cytokines by chondrocytes and synoviocytes, activates *TLR-4*, and has pro-apoptotic properties (148).

*In vivo* studies showed that a high-fat diet exacerbated OA, but n-3 PUFAs reduced disease severity by lowering inflammation and cartilage breakdown, and n-6 PUFAs had no deleterious effects on the condition (149). A diet high in eicosapentaenoic acids and docosahexaenoic acids may reduce joint stiffness and pain in individuals with arthritis (150, 151). Thus, the COX enzyme oxidizes n-6 PUFAs, producing prostaglandins (PGE2 and PGF2α), as well as leukotrienes such as LTB4. Whereas, n-3 PUFA oxidation produces less inflammatory compounds such as PGE3 and LTB5, following COX oxidation and lipooxygenase activity (150). Additionally, the study utilizing the destabilization of the medial meniscus (DMM) model demonstrates that n-3 PUFA-derived 17-hydroxy docosahexaenoic acid (17-HDHA) is linked to reduced pain during loading, confirming the murine DMM model’s utility in investigating the 17-HDHA pathway as a potential therapeutic targeting for alleviating OA pain (152).

### 3.3 Mitochondrial dysfunction

Mitochondrial dysfunction and damage, which can lead to anomalies in chondrocyte function and viability, exacerbate cartilage degradation in OA (112). These abnormalities involve the following aspects: increased inflammatory responses, such as matrix catabolism induced by interleukin-1ß *(IL-1ß*) and *TNF*, as well as impairment of chondrocyte growth and anabolic responses, excessive oxidative stress, apoptosis of chondrocytes, and calcification of the cartilage matrix (112, 119). In their study, Yun Wang (2015) revealed a dysfunction in mitochondrial biogenesis capacity, which may lead to deficits in the physiologic mitochondrial activities of chondrocytes in human knee OA (101). Thus, they connected decreased mitochondrial biogenesis capability in well-established human knee OA chondrocytes to mtDNA content and reduction in mass, as well as decreased mitochondrial function, as demonstrated through decreased baseline oxygen consumption and intracellular ATP levels (101). Multiple studies have demonstrated that mitochondrial dysfunction has an important influence on catabolic gene expression in chondrocytes. In their study, Mohammad Y (2020) demonstrated that mitochondrial roles are compromised in OA cartilage *in vivo* compared with healthy cartilage (153). Furthermore, their research demonstrated a substantial enhancement in the level of mitochondrial superoxide in human OA cartilage *in vivo*, which was associated with the expression of catabolic genes in OA cartilage. They used carbonyl cyanide 3-chlorophenylhydrazone (CCCP) to induce mitochondrial malfunction and discovered the associated signaling route. According to their findings, when CCCP-induced mitochondrial malfunction was observed *in vitro*, increases in type II collagen and proteoglycan degradation were discovered, which is consistent with the enhancement in their gene expression *in vivo*. In human and mouse cartilage explants, they also observed an enhancement in the protein expression of matrix-degrading proteases *MMP-3*, -9, -13, and *ADAMTS5* (153).

*In vivo* and *in vitro* models, by using different assessment methods such as mitochondrial DNA analysis, respiration studies, and mitochondrial membrane potential, researchers have received vital insights regarding the role of mitochondrial dysfunction in the expansion and advancement of OA. These discoveries have opened the door to more research into the underlying processes of mitochondrial dysfunction in OA, in addition to developing specialized therapeutic options for the effective treatment of this debilitating ailment.

## 4 Therapeutic implications

The search for new treatment targets is crucial, given the limited therapeutic choices. Chondrocyte metabolism appears to be an important target, given its imminent role in the pathophysiology of OA. In chondrocytes, glycolysis serves a crucial function in the setup and progression of OA. Modulating glucose metabolism may provide a novel alternative to treating OA. Therefore, glucose transporters and glycolytic enzymes could be a potential therapeutic target to modulate chondrocyte metabolism to treat OA (2). For example, icariin (ICA) is a flavonoid compound found in some plants that has been proven to have anti-inflammatory effects. ICA increases *GLUT1* and other glycolytic enzyme expression, potentially promoting anaerobic glycolysis in OA cartilage chondrocytes and enhancing cell vitality. Consequently, ICA may constitute a promising experimental treatment for OA (154). Furthermore, glucose metabolism and targeting glycolytic enzymes can influence the activity of transcription factors such as NF-κB, HIF-α, and *TGF-β*, which can influence the expression of genes involved in inflammation and cartilage breakdown. The modulation of these pathways may alleviate OA symptoms (155)..

New research has highlighted that lipid metabolism may contribute to the development and progression of OA (60). Statins have been linked to many anti-inflammatory actions in addition to their impact on lipid metabolism. They affirmed that atorvastatin, a member of the statin medication class, can prevent the development of OA (156). Statins function by inhibiting 3-hydroxy-3-methylglutaryl-coenzyme A (HMG Co-A) reductase, leading to a decrease in cholesterol levels (156). In humans, serum cholesterol levels are related to OA (84). It has been proven that intra-articular injections of statin during the development of OA reduced inflammatory cell infiltration and the expression of matrix-degrading enzymes, hence limiting cartilage deterioration (157). Statins have been found to have anti-inflammatory and chondroprotective properties in OA, reducing the generation of pro-inflammatory cytokines and improving cartilage repair (158). Resveratrol (RES) is an antioxidant that exhibits anti-inflammatory, lipid-regulating, antioxidant, and anti-aging properties (159, 160). RES has been found in animal experiments to suppress chondrocyte autophagy, apoptosis, and extracellular breakdown, resulting in a decrease in OA progression (43). In an experimental study, ChuanCai Liang (2023) found that RES has been indicated *in vivo* and *in vitro* to reduce cartilage cholesterol accumulation in OA cartilage through the intermediary of the SIRT1/foxO1 pathway, hence slowing the evolution of OA (161).

At present, there is no compound targeting mitochondria that is likely to treat OA (80). However, David (2022) proved that the gut-derived metabolite Urolithin A enhances joint mitochondrial function, reduces OA disease development, and alleviates OA pain (162). Preclinical studies suggest that mitochondrial dysfunction contributes to chondrocyte apoptosis, which can be reduced by modulating mitochondria (163). Therapeutic strategies aimed at limiting or blocking the synthesis of ROS give variable results. Two investigations, for example, have shown that vitamin C promotes apoptosis in chondrocyte cell cultures (164, 165). Hyaluronic acid is a glycosaminoglycan that is a fundamental compound of the extracellular matrix (166). It exhibits antioxidant scavenging activity against ROS/RNS and its regulatory effects are mediated by CD44 binding (166). Hyaluronic acid not only protects mtDNA damage from the initial damage caused by free radicals, but it also maintains cell viability and prevents apoptosis via the anti-CD44 antibody. Therefore, improving chondrocyte viability and maintaining mitochondrial activity under oxidative stress conditions are crucial therapeutic pathways for the effects of hyaluronic acid in OA (166). Thus, targeting mitochondrial mechanisms in chondrocytes may offer opportunities for strategies aimed at attenuating mitochondrial dysfunction in chondrocytes, which could reduce the progression of OA and protect joint function. **Table 2** summarizes different metabolic processes’ therapeutic effects, implications, and prospective therapy methods in OA.

**Table 2:** The therapeutic implication of metabolic pathways.

Process	Impact	Therapeutic implications	Treatment
Glycolysis	Increased glucose metabolism in cartilage leads to cartilage deterioration	Modulation of glucose transporters and glycolysis enzymes for cartilage protection	Icariin (128)
Lipid metabolism	Altered lipid profile in chondrocytes contributes to cartilage degradation	Modulating lipid metabolism to standardize lipid deposits and reduce inflammation	Atorvastatin (129) Resveratrol (133)
Mitochondrial dysfunction	Mitochondrial dysfunction inducing chondrocytes apoptosis and cartilage damage	Improving mitochondrial function to preserve chondrocyte’s viability and function	Urolithin A (135)

Also, some clinical trials have demonstrated significant improvements in OA symptoms by targeting chondrocyte metabolism. Metformin, an AMPK-activating medication, has shown chondroprotective actions by reducing the onset and progression of OA. Metformin works by altering mitochondria, resulting in reduced ATP synthesis. Consequently, this activates the AMPK respiratory complex I, indicating its use in clinical trials and as therapy for OA (167). Duloxetine, an effective and appropriate serotonin (5-HT) and norepinephrine (NO) reuptake inhibitor (SNRI), is used to treat severe depression, anxiety, diabetic nerve damage, and fibromyalgia. In recent trials, duloxetine at a dose of 60/120 mg per day reduced the discomfort of individuals with knee OA (168).

One proposed strategy is to enhance the pharmacological effects of β-Caryophyllene (BCP), a plant-derived sesquiterpene that interacts with cannabinoid receptor (CB) 2 and exhibits anti-inflammatory properties by reducing *MMPs* and *IL-1β* production in human chondrocytes. The combination of BCP with antioxidants, such as ascorbic acid (AA), and chondro-protective elements, such as GlcN, increases the production of proteoglycans and has anti-inflammatory and anticatabolic effects. Additionally, AA’s antioxidant properties can help minimize oxidative stress linked to OA progression by inducing proteoglycan generation in chondrocytes (169).

Physical activity is favorably recommended by the American College of Rheumatology for improving OA incomes and reducing disability (170). Regular exercise may enhance OA outcomes by aiding in weight loss and decreasing joint loading (171), decreasing systematic inflammatory biomarkers (such as *IL-6* associated with cartilage degradation) (172), and providing appropriate loading of articular cartilage, which is essential for preserving tissue integrity (173).

Research also indicates that healthy dietary regimes and nutrition interventions ameliorate OA progression (174), reduce inflammatory markers that accelerate cartilage metabolism (175), and lead to decreases in body weight (176). Based on studies, adhering to Mediterranean-type diets can mitigate the severity and progression of OA, as demonstrated by enhancements in outcomes reported by patients, such as degrees of pain, movement, symptoms, cartilage deterioration, and inflammatory biomarkers (177, 178). While Mediterranean-type diets may differ, they usually include a variety of fruits, vegetables, legumes, nuts, and seafood, with a moderate intake of dairy, olive oil, and poultry (179). Certain foods, such as ginger and strawberries, may enhance symptom relief via antioxidant mechanisms, which can have negative effects on nearby tissues and promote inflammation (180).

## 5 Discussion

Our investigation in this review focused on the *in vivo* and *in vitro* metabolic properties and alterations of chondrocytes when exposed to OA. We highlighted the complex interplay between metabolic pathways, such as glycolysis, lipid metabolism, and mitochondrial function. Dysregulation in certain metabolic pathways, such as glycolysis, lipid metabolism, and mitochondrial activity, contributes significantly to the pathogenesis of OA. Glycolysis, the mechanism through which glucose is broken down to produce energy in the form of ATP, has been related to OA pathogenesis. Increased glycolytic activity in chondrocytes can cause an increase in ROS generation (40). Excessive ROS can cause oxidative stress and damage to biological compounds such as mitochondria (112–114). Altered lipid metabolism in OA induces lipid buildup in chondrocytes, causing cell stress and dysfunction. This lipid accumulation causes dysfunction in mitochondria, impairs the metabolism of lipids, and alters lipid balance. Therefore, this imbalance promotes oxidative stress in chondrocytes, exacerbating the deterioration in OA-affected cartilage (74). Mitochondrial failure in OA affects cellular metabolism, resulting in decreased ATP synthesis, increased ROS generation, and altered signaling cascades (29, 101). This malfunction can promote chondrocyte death and inflammation, which can eventually lead to cartilage breakdown in OA.

*In vivo* studies provided insights into the metabolic changes of chondrocytes in OA. Increased proteoglycan degradation has been shown in high-glucose milieus. In lipidemia, high expression of proteolytic enzymes was seen, and that was followed by a decrease in collagen type II protein levels, further aggravating the metabolic imbalance in OA.

*In vitro* studies, an excessive production of ROS was found in glucose update assessments. An increase in type II collagen and proteoglycan degradation was noted in mitochondrial-induced malfunction. Furthermore, the matrix-degrading proteases *MMP-3*, *-9*, *-13*, and *ADAMTS5* had increased protein expression in human and mouse cartilage explants (153). The studies also suggested that there is expression and release of pro-inflammatory mediators in OA, which further leads to disease progression.

The interaction between these pathways remains unclear, but all of these metabolic changes are interconnected and may contribute to cartilage degeneration and OA progression. For instance, enhanced glycolytic activity can produce ROS (40), which can promote mitochondrial dysfunction (112, 114). Altered lipid metabolism could also contribute to mitochondrial dysfunction and increased oxidative stress in chondrocytes (71). Overall, these metabolic alterations interact to disrupt chondrocyte functions, promote inflammation, and accelerate cartilage degeneration in OA.

These metabolic changes in OA chondrocytes can be potential strategies to treat OA. For example, icariin (ICA) increases *GLUT1* expression and other glycolytic enzymes, potentially promoting anaerobic glycolysis in OA cartilage chondrocytes and enhancing cell vitality (154). Furthermore, physical modalities and diets may be effective methods for managing symptoms and decelerating disease development by modulating chondrocyte metabolism.

The interaction between different processes is considered an important limitation of the study. Additionally, the challenge of this study is that *in vivo* and *in vitro* models may not fully reproduce the disease’s complex and dynamic character as it occurs in the human body. *In vivo* studies may be constrained by variations in genetic origins, environmental circumstances, and disease progression in animal models and humans. Also, *in vitro* studies may not fully capture the relationships and effects of various cell types, tissues, and signaling pathways that contribute to OA in the joint environment. These limitations make it difficult to precisely analyze metabolic processes and anticipate the effects of therapy in trials.

In the future, we will study the different interactions between metabolic pathways and chondrocytes, such as glycolysis, lipid metabolism, and mitochondrial function, to gain an extensive understanding of the influence of metabolic mechanisms on OA etiology. We need to observe and assess the metabolic activity of chondrocytes, which will give us a dynamic view of metabolic alterations during OA. Finally, we need to identify new chondrocyte metabolic targets for potential therapy to modulate chondrocyte metabolism.

## 6 Conclusion

This study provided insight into how chondrocytes can undergo various metabolic changes during OA, both *in vivo* and *in vitro*. These changes include mitochondrial dysfunction and a shift towards glycolysis, which are related to chondrocyte catabolism and cartilage deterioration. Ultimately, the results of *in vivo* and *in vitro* investigations imply that metabolic alterations are essential to the etiology of OA. Targeting these metabolic changes may offer new alternative options for OA treatment. Future research should also focus on the interaction between the different metabolic pathways in chondrocytes and their alterations during OA, as well as the strategies to treat OA by targeting these metabolic pathways.

## Author contributions

MS: Writing – review & editing, Writing – original draft. HZ: Writing – review & editing. XR: Writing – review & editing. YZ: Writing – review & editing. PZ: Writing – review & editing, Funding acquisition.
